# Physiology

**Published:** 1876-08

**Authors:** J. F. Sanborn


DENTAL REGISTER.

VOL. XXX.] AUGUST, 1876. [No. 8.

### PHYSIOLOGY.

BY J. F. SANBORN.

*Read before the Iowa State Dental Society.*

Mr. President and members of the Iowa State Dental Society:—In looking about for a subject to instruct and entertain you for an hour, in matters that every one should be more or less learned in, the more the better, and in which ignorance is not bliss, neither is it folly to be wise, I have selected "Physiology and Hygiene."

My subject covers a great deal of ground, in fact enough for a whole course of lectures, each one presenting some important physiological principal. I shall therefore present but little more than the facts and principles, omitting the reasons that lead to such conclusions.

What constitutes the vital principle I do not claim to be able to define; but that vital principle in its connection with the matter that constitutes the body, has certain manifestations in which its organs perform their functions, the performing of which is essential to the continuance of the vital principle in connection with the body which it animates.

This active condition is known as life. Life, then, is action; the cessation of that action is death.

The various actions that go to make up the sum of life may be classed under four heads:

1. Alimentation, which embraces all the changes from food received, till the nutritious portion of it is introduced into the blood.

2. Assimilation, embracing the changes of the nutritious matter in the blood into the various tissues of the body.

3. Disintegration, which is the breaking down of the cell structure.

4. Depurition, which is the vital action of removing broken down and refuse matter from the system.

Health is the perfect balance in these four classes of actions. Alimentation and assimilation constitute the build up side of vital actions; disintegration and depurition, the take down side of such actions. Life is an active, positive condition; death is an inactive or negative condition. Health is the perfect balance in these vital actions. Disease is nature's effort to restore the balance when it is lost. Alimentation, embraces prehension, mastication, insalivation, dilution, digestion, and absorption. Digestion has for its object the reducing of the food eaten to a semi-fluid condition and also to aid in a series of transformations by which these principles are rendered capable of nourishing the organism. These principles thus prepared are absorbed and pass into the circulation as fast as the requisite changes in their constituents are effected, and there become a part of the great nutritive fluid supplying the waste which the constant regeneration of the tissues from materials furnished by the blood necessarily involves. The digestion of oleaginous and fatty ingesta, are an exception to this general law. When it is borne in mind that all nutriment must be reduced to a semi-fluid condition before it can be absorbed, it will be seen that thorough mastication greatly facilitates this operation. Good teeth are essential to thorough mastication. "Pretty is as pretty does" applies to the results of our operations, as in any other branch of human industry. Food then should be well masticated, that it may be more easily digested and reduced to the semi-fluid condition; and also that it may be well mixed with the fluids of the mouth. No other fluid will so well perform the great end demanded, as the saliva, so that those who swallow their food half masticated, and float it down with tea or coffee, water, milk or any other fluid, do so in violation of this well known physical law, and they must sooner or later suffer the penalty—dyspepsia. This disease is not followed by immediate death, but the vital powers are more or less deranged, according to the amount of vital force that the person may possess; the standard of vitality, is lowered, that beautiful balance that constitutes health has been lost, and the cessation of those vital changes that constitute life will sooner remove him from his sphere of usefulness, than if the laws of vitality had been observed.

The talent that God has given to our keeping, is often laid up in a napkin, and the usefulness we might otherwise exert is lost to the world, on account of disease which is the direct result of this violation of the laws of life. Physical sins are not forgiven; God is not mocked when we apply the break to the wheels of life.

The food in the stomach is passed from one part of it to another and becomes mixed with the gastric juice, and as far as possible is reduced to a semi-fluid condition. The part of the food known as nitrogenous, that is, the lean part of the meat, the glutinous portions that surrounds the fat particles in fat meat, the gluten of bread or the part of it that nourishes the muscular tissues, these are digested in the stomach and reduced to a pultaceous fluid which passes into the small intestines, where the process is completed. Liquids and a part of the before described ingesta are absorbed from the stomach and pass directly into the circulation. Sugar cooked starch or dextrine, and farinaceous preparations generally, fruit and vegetables are not effected by the gastric juice, but pass to the intestines, there to be acted on and digested by the juices there found. The gastric juice, when freed from mucus and other foreign matter, is a clear fluid of a faint yellowish, or amber color, with little or no vicidity. Its reaction is strongly acid. The digestive principle is called pepsin. "Pepsin is soluble in water, but is precipitated by alcohol." When dried in thin slices on a plate of glass it is in the form of small, grayish, translucent scales, with but little smell, and a feeble, bitter, nauseous taste.

The experiments of Dr. Beaumont in 1823 on Alex. St. Martin, furnish the most satisfactory information in regard to the characteristics of the gastric juice; the digestive principle it contains, pepsin, and the length of time required to digest various articles of food.

From three to three and a half hours is about the average length of time required for stomach digestion. After the stomach has performed its duty on the ingesta, it is passed to the duodenum for the completion of the digestive process. It is supplied with a double class of vessels; first, the glands that supply the juices that complete the digestive process, and second, the villi that absorb the nutriment and convey it into the circulation as fast as it is prepared. Both of these actions are going on simultaneously.

The intestinal juice digests sugar, starch, dextrine, fruit, and vegetables, and completes that began in the stomach on lean meat, gluten of the cereals, casein of milk, and other nitrogenous ingesta. This class of nutriment, as fast as it becomes fully prepared, is absorbed by the veins of the villi and is conveyed by the veins of the portal system to the liver and from there to the circulation. The villi are from forty to ninety to the square inch of surface, and aggregate 4,000,000. In addition to their duties as just described, they act as sentinels to guard the entrance to the citadel of life against such enemies as have passed the picket guards. Many of the poisons which would cause certain death if introduced into the circulation, by innoculation, can be taken in the stomach in a hundred times the quantity, and, thanks to these life guards, pass outward without material injury.

Food may be divided into nitrogenous—as lean meats, the white of eggs, and the like—non-nitrogenous, or, as I prefer to call them, hydro-carbonaceous—such as starch, dextrine, cane sugar, and fat and other oleaginous substances. They are, chemically, $C_{12}H_{10}O_{10}$ or more, and are nearly allied to each other, and have so small a proportion of oxygen that they remain for a long time at ordinary temperature without material change. Acid fruits are abundantly supplied with free oxygen—that is, oxygen not chemically combined with both ingredients. Such fruits soon decay. This fact is well understood by the good housewife without understanding why it is so. So she mixes hydro-carbon of sugar with equal parts of fruit, and the compound will keep for months or years under favorable circumstances.

The liver is, so to speak, the safety-valve to the system, to regulate the hydro-carbons as they enter the system, with the exception of fat, which passes into the circulation by way of the thoracic duct, and is found deposited in the various parts of the system. The sugar and starches pass with the nitrogenous food through the liver, and the bile, which is a hydro-carbon, is eliminated. An excessive supply of the hydro-carbon, in our food in proportion to the nitrogenous, and without the free oxygen fruits to restore the balance, is the primary cause of an enlarged liver, congested liver, or in plain English, an over worked liver, and as a result, the excess of the hydro-carbon is carried into the circulation and causes jaundice, biliousness, and the effort of vitality to restore the balance occasions a fever, and the various symptoms that accompany the fever have given the various names to it. If it is desired to have this condition of things so mystified that no one can see it, then call it miasm that has caused it. So in the midst of the miasm, your attention is called to the digestion of the hydro-carbon fat and its congeners. The fat of fat meat, is finely divided molecules enveloped in fibrous tissues; and when eaten the stomach by digestion removes the covering from the molecules, and sets the fat free, which passcs unchanged till it comes in contact with the bile and pancreatic juice, which mixing with it forms an emulsion by finely dividing the fat into very minute particles; this emulsion is absorbed by the lacteals and is passed through the mesenteric glands on its way to the thoracic duct, through which it passes to the left sub-clavian vein, where it enters the circulation.

The amount of nutriment that is absorbed and passed into the circulation, under ordinary natural alimentation, is nine-tenths of the food eaten; the one-tenth is passed as refuse matter.

In over-feeding, as in fattening animals, a very much smaller per cent is used, and it is a fact well known to agriculturalists that the droppings of stall-fed animals is vastly richer in plant food than that of other stock. As has been stated, the food that nourishes the muscles and like tissues, is known as nitrogenous; that which forms adipose tissue, such as sugar, starch, dextrine, fat, butter and the like, are hydro-carbons, and when analyzed are found to be composed of the same ingredients in but slightly different proportions.

In digesting, starch is changed to dextrine, which is then soluble in water. Starch is not, until by cooking, it is changed to dextrine. Dextrine is changed to glucose, or grape sugar; cane sugar is changed to grape sugar, and then is absorbed. It will be seen by these transformations of starch to dextrine, dextrine to cane sugar, cane sugar to grape sugar, that the grape is peculiarly adapted to the wants of vitality, in that it contains the glucose in the condition or state of development that it can be used with but little, if any, change, except simple digestion. The grape is also an acid fruit, and contains free oxygen which is a counterpoise to the hydro-carbons which neither of the hydro-carbons possess. This is the reason why the grape is so peculiarly adapted, not only as a preventive, but a panacea for all the various bilious diseases. Fat is not materially changed in the process of digestion. It is found as fat in the blood and continues such as it passes through the various parts of the circulation till it becomes entangled in the meshes of the tissues, where it remains until reabsorbed into the blood and is elminated through the agency of the liver, lungs and skin, and in the order named. Water performs so very important a part in the vital changes that it demands more than a passing notice. All the various kinds of food which have received our attention are organic matter, that is, they are the result of growth, either vegetable or animal, and are changed in passing through the digestive process to which they are subject; but water is an inorganic fluid, and is not changed, it is not digested, but passes readily from the stomach to the blood, and from the blood through the glands to the digestive apparatus; it passes either way according to the demands of the case, in any part of the system. It is used in liquifying the solids to a condition so as to be readily carried through the fine capillaries to where they are wanted to build up the tissues. In this manner it is essential to the digestion and the circulation. Its second, and no less important use, is to dissolve out the solids that have subserved their day and become broken down matter by reducing them to a fluid condition, so that they can be removed. On the ocean, lakes, rivers and canals it has its use as a medium of intercommunication, to facilitate the carriage of commerce to and fro through the length and breadth of the world; as on the Erie and other canals, so it is in the alimentary canal; it is used as a medium of intercommunication through various parts of our body. It also subserves a very important part in regulating the temperature of our bodies by evaporation. Water bears as perfect a relationship to the wants of vitality as a wise God in His beneficence and wisdom can make it. It is one of the greatest and most precious gifts that he has bestowed on a fallen world. Without it our beautiful world would be a desert devoid of vegetation and animal life, a mass of elements without use, being blown about by every passing wind. With it we have this beautiful world with all its mighty commerce, its busy millions with all their cares, joys and sorrows.

Water is a fluid prepared by God himself to invigorate His creatures and beautify his footstool, and so perfectly adapted to our wants that our first parents might as well have thought of adding to the bliss of paradise by partaking of the forbidden fruit as to enhance the adaptibility of water to their physical wants by adding to it aught that their ingenuity could have devised to an unnatural appetite.

A prize fighter when being trained for a "mill," as it is called, desired a small portion of wine or beer a day, was answered: "Then you must get some one else to train you." In prize fighting it is necessary to develop the vital powers to the highest degree possible. We may learn from this that the highest physical development is incompatible with the use of such drinks. Assimilation, or the second class of vital actions, is the changing of the plasma in the blood into the various tissues of the body. This action takes place only during sleep. Sleep is the suspension of all action of the wakeful hours and the direction of the vital energies to the reconstruction and repair wherever it is wanted. Digestion of food should always take place during wakeful hours. The break down of tissue nearly all of it takes place during wakeful hours. But the build up of tissue takes place only during sleep. The tissues when developed subserve the end for which they were designed by the Great Architect of the universe, "each in its proper station moves, and each fulfills its part."

We are continually breathing oxygen from the sourrounding air. This is conveyed in the red corpuscles of the blood to every part of the body, and is there ready provided, wherever and whenever wanted, to unite with the tissues, disintegrating them or reducing them to a broken down condition. Oxygen is a universal breakdown agent, and not a build up agent. The break-down of tissue is as much a vital process as is the build up of tissue, and oxygen is a "vital air," in that it performs this important function.

The fourth class of actions is the removal of the broken down, waste matter from the system and is known as depuration. The lower bowels, kidneys, lungs, liver and skin, perform this duty, and in order to perfect health, each should perform its whole duty, and so long as they do perform their various functions, and the circulation is kept well balanced, health will be maintained; and the golden key to the success of physiological treatment of disease, is to balance the circulation, and see that each of the emunctories are in balance, or in other words nature's demand is true obedience to physiological law.

The length of this essay is such that the society must excuse me from the second part of our subject, which will be a good subject for a future essay.

